# Supplementary material for: Type-I myosins promote actin polymerization to drive membrane bending in endocytosis
Source: eLife. 2019 Aug 6;8:e44215. doi: 10.7554/eLife.44215 (PMC6684269; doi:10.7554/eLife.44215)
Supplement: Supplementary file 1. — Supplemental table 1: Sla1 centroid speeds per genotype. Supplemental table 2: Myosin molecule numbers per genotype. Supplemental table 3: Yeast strains used in this work. Supplemental table 4: Plasmids used in this work. Supplemental table 5: Number of trajectories per dataset. Supplemental table 6: Number of analyzed endocytic sites for quantification of molecule numbers. Supplemental table 7: Summary of CLEM data. [file elife-44215-supp1.docx]

# Supplemental tables

- Supplemental table 1: Sla1 centroid speeds per genotype
- Supplemental table 2: Myosin molecule numbers per genotype
- Supplemental table 3: Yeast strains used in this work
- Supplemental table 4: Plasmids used in this work
- Supplemental table 5: Number of trajectories per dataset
- Supplemental table 6: Number of analyzed endocytic sites for quantification of molecule numbers
- Supplemental table 7: Summary of CLEM data

|  | **Combined datasets** | | | **Replicate 1** | | | **Replicate 2** | | |  |
| --- | --- | --- | --- | --- | --- | --- | --- | --- | --- | --- |
| **Genotype** | **Mean  (nm/s)** | **SE** | **n** | **Mean  (nm/s)** | **SE** | **n** | **Mean  (nm/s)** | **SE** | **n** |  |
| WT | **26.2** | 1.38 | 54 | 25.44 | 1.96 | 31 | 27.13 | 1.91 | 24 | haploids |
| *myo3Δ* | **24.1** | 1.15 | 72 | 24.17 | 1.48 | 32 | 24.08 | 1.71 | 40 |  |
| *myo5Δ* | **17.1** | 1.41 | 76 | 16.46 | 2.46 | 25 | 17.81 | 1.81 | 21 |  |
| *myo5-CAΔ* | **26.1** | 1.34 | 52 | 23.88 | 1.62 | 24 | 27.97 | 2.02 | 28 |  |
| *MYO5 dupl.* | **30.6** | 1.05 | 64 | 29.20 | 1.55 | 31 | 31.87 | 1.41 | 33 |  |
| *bbc1Δ* |  |  |  | 29.53 | 2.22 | 20 |  |  |  |  |
| *myo5Δ bbc1Δ* |  |  |  | 16.28 | 1.73 | 20 |  |  |  |  |
| *WT/WT* | **25.2** | 0.94 | 82 | 25.42 | 1.32 | 48 | 24.97 | 1.30 | 34 | diploids |
| *MYO5/myo5Δ* | **23.7** | 0.72 | 86 | 23.48 | 1.01 | 44 | 23.84 | 1.03 | 42 |  |
| *myo5Δ/myo5Δ* | **13.9** | 1.04 | 74 | 12.83 | 1.20 | 38 | 15.07 | 1.72 | 36 |  |

## Supplemental table 1: Sla1 inward movement rates in different genetic backgrounds.

To calculate the Sla1 inward movement speed, a linear regression was fitted through a 2 s period corresponding to the inward movement phase of each individual trajectory. (see materials and methods). For most genotypes, two independent datasets were collected, tested whether they were the same, and subsequently pooled. SE = standard error, n = number of trajectories fitted.

| **Protein** | **Myo3-EGFP** | | **Myo5-EGFP** | | **Total myosin** | |  |
| --- | --- | --- | --- | --- | --- | --- | --- |
| **Genotype** | **nr mols** | **SEM** | **nr mols** | **SEM** | **nr mols** | **SEM** |  |
| WT | 37.8 | 4.0 | 76.7 | 8.1 | 114.5 | 9.0 | haploids |
| *myo3Δ* | x | x | 87.2 | 9.6 | 87.2 | 9.6 |  |
| *myo5Δ* | 34.1 | 3.8 | x | x | 34.1 | 3.8 |  |
| *MYO5 dupl.* | 40.1 | 4.4 | 111.4 | 11.9 | 151.5 | 12.7 |  |
| *MYO5 dupl., myo3Δ* | x | x | 156.3 | 16.8 | 156.3 | 16.8 |  |
| *MYO3 dupl.* | 59.5 | 6.3 | 74.9 | 8.0 | 134.4 | 10.2 |  |
| *MYO3 dupl., myo5Δ* | 56.7 | 6.1 | x | x | 56.7 | 6.1 |  |
| *Myo5-G132R* | ND | ND | 44.4 | 4.6 | ND | ND |  |
| WT/WT | 35.1 | 3.8 | 73.5 | 7.6 | 108.6 | 8.5 | diploids |
| MYO5/myo5Δ | 36.3 | 3.9 | 58.0 | 6.7 | 94.3 | 7.8 |  |
| myo5Δ/myo5Δ | 34.9 | 3.8 | x | x | 34.9 | 3.8 |  |
| MYO3/myo3Δ, myo5Δ/myo5Δ | 14.4 | 1.6 | x | x | 14.4 | 1.6 |  |

## Supplemental table 2: Myosin-I molecule numbers per endocytic site in different genetic backgrounds.

Median number of molecules over the lifetime of the patch and SEM for Myo3-EGFP and Myo5-EGFP in cells with different genetic backgrounds. “Total myosin” gives the sum of Myo3 and Myo5. x = cannot be measured, ND = not determined.

## Supplemental table 3: Yeast strains used in this work

| **Name** | **Genotype** |
| --- | --- |
| **MKY0100** | MATa, his3-Δ200, leu2-3,112, ura3-52, lys2-801 |
| **MKY0102** | MATα, his3-Δ200, leu2-3,112, ura3-52, lys2-801 |
| **MKY0105** | MATa/MATα, his3-Δ200/his3-Δ200, leu2-3,112/leu2-3,112, ura3-52/ura3-52, lys2-801/lys2-801 |
| **MKY0216** | MATa, his3-Δ200, leu2-3,112, ura3-52, lys2-801, NUF2-EGFP::his3MX6 |
| **MKY0217** | MATα, his3-Δ200, leu2-3,112, ura3-52, lys2-801, NUF2-EGFP::his3MX6 |
| **MKY0224** | MATa, his3-Δ200, leu2-3,112, ura3-52, lys2-801, NUF2-mCherry::KANMX4 |
| **MKY2653** | MATa, his3-Δ200, leu2-3,112, ura3-52, lys2-801 with pMK0100 [CEN, URA3 GFP-ACT1] |
| **MKY2654** | MATα, his3-Δ200, leu2-3,112, ura3-52, lys2-801 with pMK0100 [CEN, URA3 GFP-ACT1] |
| **MKY2655** | MATα, his3-Δ200, leu2-3,112, ura3-52, lys2-801, ABP1-mCherry::kanMX4 with pMK0100 [CEN, URA3 GFP-ACT1] |
| **MKY2689** | MATα, his3-Δ200, GalL-ISce1-nat::leu2-3,112, ura3-52, lys2-801, sfGFP-SLA2 |
| **MKY2720** | MATa, his3-Δ200, leu2-3,112, ura3-52, lys2-801, ARC18-myEGFP::natNT2, ABP1-mCherry::kanMX4 |
| **MKY2747** | MATa, his3-Δ200, leu2-3,112, ura3-52, lys2-801, ARC18-myEGFP::natNT2 |
| **MKY2832** | MATa, his3-Δ200, leu2-3,112, ura3-52, lys2-801, RVS167-EGFP::his3MX6 |
| **MKY2833** | MATa, his3-Δ200, leu2-3,112, ura3-52, lys2-801, SLA1-EGFP:his3MX6 |
| **MKY2834** | MATα, his3-Δ200, leu2-3,112, ura3-52, lys2-801, ABP1-EGFP::his3MX6 |
| **MKY2836** | MATα, his3-Δ200, ura3-52, lys2-801, GalL-ISce1-nat::leu2-3,112, sfGFP-Sla2, ABP1-mCherry::kanMX4 |
| **MKY2865** | MATa, his3-Δ200, leu2-3,112, ura3-52, lys2-801, CAP1-EGFP::his3MX6 |
| **MKY2876** | MATa, his3-Δ200 leu2-3,112, ura3-52, lys2-801, MYO5-EGFP::his3MX6 |
| **MKY2920** | MATα, his3-Δ200, leu2-3,112, ura3-52, lys2-801, NUF2-sfGFP::his3MX6 |
| **MKY3074** | MATα, his3-Δ200, leu2-3,112, ura3-52, lys2-801, ABP1-mCherry::kanMX4, SLA1-EGFP::his3MX6 |
| **MKY3131** | MATa, his3-Δ200, leu2-3,112, ura3-52, lys2-801, ABP1-mCherry::kanMX4, RVS167-EGFP::his3MX6 |
| **MKY3151** | MATa, his3-Δ200, leu2-3,112, ura3-52, lys2-801, MYO5-EGFP::his3MX6, ABP1-mCherry::kanMX4 |
| **MKY3166** | MATa, his3-Δ200, leu2-3,112, ura3-52, lys2-801, SLA1-EGFP::his3MX6, myo5∆::natNT2 |
| **MKY3168** | MATa, his3-Δ200, leu2-3,112, ura3-52, lys2-801, SLA1-EGFP::his3MX6, ABP1-mCherry::kanMX4, myo5∆::natNT2 |
| **MKY3173** | MATa/α, his3-Δ200/his3-Δ200, leu2-3,112/leu2-3,112, ura3-52/ura3-52, lys2-801/lys2-801, SLA1-EGFP::his3MX6/SLA1-EGFP::his3MX6 |
| **MKY3175** | MATa/α, his3-Δ200/his3-Δ200, leu2-3,112/leu2-3,112, ura3-52/ura3-52, lys2-801/lys2-801, SLA1-EGFP::his3MX6/SLA1-EGFP::his3MX6, myo5∆::natNT2/myo5∆::natNT2 |
| **MKY3186** | MATa/α, his3-Δ200/his3-Δ200, leu2-3,112/leu2-3,112, ura3-52/ura3-52, lys2-801/lys2-801, SLA1-EGFP::his3MX6/SLA1-EGFP::his3MX6, MYO5/myo5∆::natNT2 |
| **MKY3188** | MATa, his3-Δ200, leu2-3,112, ura3-52, lys2-801, ABP1-EGFP::his3MX6, myo5∆::natNT2 |
| **MKY3196** | MATα, his3-Δ200, leu2-3,112, ura3-52, lys2-801, ABP1-EGFP::his3MX6, myo3∆::natNT2 |
| **MKY3216** | MATa, his3-Δ200, leu2-3,112, ura3-52, lys2-801, SLA1-EGFP::his3MX6, myo3∆::natNT2 |
| **MKY3223** | MATa/α, his3-Δ200/his3-Δ200, leu2-3,112/leu2-3,112, ura3-52/ura3-52, lys2-801/lys2-801, myo3∆::natNT2/myo3∆::natNT2, SLA1-EGFP::his3MX6/SLA1-EGFP::his3MX6 |
| **MKY3226** | MATa/α, his3-Δ200/his3-Δ200, leu2-3,112/leu2-3,112, ura3-52/ura3-52, lys2-801/lys2-801, ABP1-EGFP::his3MX6/ABP1-EGFP::his3MX6 |
| **MKY3227** | MATa, his3-Δ200, leu2-3,112, ura3-52, lys2-801, RVS167-EGFP::his3MX6, myo5∆::natNT2 |
| **MKY3228** | MATα, his3-Δ200, leu2-3,112, ura3-52, lys2-801, RVS167-EGFP::his3MX6, myo5∆::natNT2 |
| **MKY3229** | MATa, his3-Δ200, leu2-3,112, ura3-52, lys2-801, ABP1-mCherry::kanMX4, RVS167-EGFP::his3MX6, myo5∆::natNT2 |
| **MKY3244** | MATa, his3-Δ200, leu2-3,112, ura3-52, lys2-801, MYO5-EGFP::his3MX6, ABP1-mCherry::kanMX4, myo3∆::natNT2 |
| **MKY3318** | MATα, his3-Δ200, leu2-3,112, ura3-52, lys2-801, MYO3-EGFP::his3MX6 |
| **MKY3340** | MATα, his3-Δ200, leu2-3,112, ura3-52, lys2-801, MYO3-EGFP::his3MX6, myo5∆::natNT2 |
| **MKY3355** | MATa, his3-Δ200, leu2-3,112, ura3-52, lys2-801, SLA1-EGFP::his3MX6, myo5-CA∆::natNT2 |
| **MKY3403** | MATa, his3-Δ200, leu2-3,112, ura3-52, lys2-801, SLA1-EGFP::his3MX6, myo5∆::natNT2, myo3∆::natNT2 |
| **MKY3405** | MATa, his3-Δ200, leu2-3,112, ura3-52, lys2-801, SLA1-EGFP::his3MX6, myo5∆::natNT2, myo3∆::natNT2, ABP1-mCherry::kanMX4 |
| **MKY3412** | MATa, his3-Δ200, leu2-3,112, ura3-52, lys2-801, SLA1-EGFP::his3MX6, bbc1∆::natNT2 |
| **MKY3459** | MATα, his3-Δ200, leu2-3,112, ura3-52, lys2-801, MYO3-EGFP::his3MX6, ABP1-mCherry::kanMX4 |
| **MKY3508** | MATa, his3-Δ200, leu2-3,112, ura3-52, lys2-801, SLA1-EGFP::his3MX6, MYO5::hphNT1::MYO5 |
| **MKY3512** | MATa/α, his3-Δ200/his3-Δ200, leu2-3,112/leu2-3,112, ura3-52/ura3-52, lys2-801/lys2-801, MYO3-EGFP::his3MX6/MYO3-EGFP::his3MX6 |
| **MKY3513** | MATa/α, his3-Δ200/his3-Δ200, leu2-3,112/leu2-3,112, ura3-52/ura3-52, lys2-801/lys2-801, MYO3-EGFP::his3MX6/MYO3-EGFP::his3MX6, MYO5/myo5∆::natNT2 |
| **MKY3514** | MATa/α, his3-Δ200/his3-Δ200, leu2-3,112/leu2-3,112, ura3-52/ura3-52, lys2-801/lys2-801, MYO3-EGFP::his3MX6/MYO3-EGFP::his3MX6, myo5∆::natNT2/myo5∆::natNT2 |
| **MKY3526** | MATa/α, his3-Δ200/his3-Δ200, leu2-3,112/leu2-3,112, ura3-52/ura3-52, lys2-801/lys2-801, myo3∆::hphNT1/MYO3-EGFP::his3MX6, myo5∆::natNT2/myo5∆::natNT2 |
| **MKY3533** | MATa, his3-Δ200, leu2-3,112, ura3-52, lys2-801, MYO5-EGFP::his3MX6::hphNT1::MYO5-EGFP::his3MX6 |
| **MKY3535** | MATα, his3-Δ200, leu2-3,112, ura3-52, lys2-801, MYO3-EGFP::his3MX6, MYO5::hphNT1::MYO5 |
| **MKY3536** | MATa/α, his3-Δ200/his3-Δ200, leu2-3,112/leu2-3,112, ura3-52/ura3-52, lys2-801/lys2-801, myo3∆::natNT2/myo3∆::natNT2, MYO5-EGFP::his3MX6/myo5∆::hphNT1 |
| **MKY3537** | MATa, his3-Δ200, leu2-3,112, ura3-52, lys2-801, SLA1-EGFP::his3MX6, MYO3::hphNT1::MYO3 |
| **MKY3556** | MATa/α, his3-Δ200/his3-Δ200, leu2-3,112/leu2-3,112, ura3-52/ura3-52, lys2-801/lys2-801, myo3∆::natNT2/myo3∆::natNT2, MYO5/myo5∆::hphNT1, SLA1-EGFP::his3MX6/SLA1-EGFP::his3MX6 |
| **MKY3565** | MATα, his3-Δ200, leu2-3,112, ura3-52, lys2-801, MYO3-EGFP::his3MX6::hphNT1::MYO3-EGFP::his3MX6, myo5∆::natNT2 |
| **MKY3590** | MATa, his3-Δ200, leu2-3,112, ura3-52, lys2-801, myo5∆::natNT2 with pMK0100[CEN, URA3 GFP-ACT1] |
| **MKY3616** | MATα, his3-Δ200, leu2-3,112, ura3-52, lys2-801, sfGFP-SLA2, myo5∆::natNT2 |
| **MKY3631** | MATa, his3-Δ200, ura3-52, lys2-801, sfGFP-SLA2, myo5∆::natNT2, ABP1-mCherry::kanMX4, and GalL-ISce1-NAT::leu2-3,112 or leu2-3,112 |
| **MKY3633** | MATa, his3-Δ200, leu2-3,112, ura3-52, lys2-801, MYO5-EGFP::his3MX6MX6, MYO3::hphNT1::MYO3 |
| **MKY3634** | MATα, his3-Δ200, leu2-3,112, ura3-52, lys2-801, SLA1-EGFP::his3MX6, myo5∆::natNT2, MYO3-EGFP::his3MX6::hphNT1::MYO3-EGFP::his3MX6 |
| **MKY3656** | MATa, his3-Δ200, leu2-3,112, ura3-52, lys2-801, CAP1-EGFP::his3MX6, myo5∆::natNT2 |
| **MKY3658** | MATa, his3-Δ200, leu2-3,112, ura3-52, lys2-801, CAP1-EGFP::his3MX6, myo5∆::natNT2, ABP1-mCherry::kanMX4 |
| **MKY3660** | MATa, his3-Δ200, leu2-3,112, ura3-52, lys2-801, CAP1-EGFP::his3MX6, ABP1-mCherry::kanMX4 |
| **MKY3665** | MATa, his3-Δ200, leu2-3,112, ura3-52, lys2-801, myo5∆::natNT2, ARC18-myEGFP::natNT2 |
| **MKY3667** | MATa, his3-Δ200, leu2-3,112, ura3-52, lys2-801, myo5∆::natNT2, ARC18-myEGFP::natNT2, ABP1-mCherry::kanMX4 |
| **MKY3733** | MATa, his3-Δ200, leu2-3,112, ura3-52, lys2-801, myo5-CA∆::NAT, ARC18-myEGFP::natNT2 |
| **MKY3754** | MATa, his3-Δ200, leu2-3,112, ura3-52, lys2-801, myo5-CA∆::natNT2 with pMK0100[CEN, URA3 GFP-ACT1] |
| **MKY3815** | MATa, his3-Δ200, leu2-3,112, ura3-52, lys2-801, SLA1-EGFP::his3MX6, myo5∆::natNT2, bbc1∆::natNT2 |
| **MKY3816** | MATα, his3-Δ200, leu2-3,112, ura3-52, lys2-801, SLA1-EGFP::his3MX6, myo5∆::natNT2, bbc1∆::natNT2 |
| **MKY3819** | MATa, his3-Δ200, leu2-3,112, ura3-52, lys2-801, myo5∆::natNT2, bbc1∆::natNT2 with pMK0100[CEN, URA3 GFP-ACT1] |
| **MKY3863** | MATα, his3-Δ200, leu2-3,112, ura3-52, lys2-801, SAC6-EGFP::his3MX6 |
| **MKY3865** | MATa, his3-Δ200, leu2-3,112, ura3-52, lys2-801, SAC6-EGFP::his3MX6, ABP1-mCherry::kanMX4 |
| **MKY3867** | MATa, his3-Δ200, leu2-3,112, ura3-52, lys2-801, myo5∆::natNT2, SAC6-EGFP::his3MX6 |
| **MKY3868** | MATa, his3-Δ200, leu2-3,112, ura3-52, lys2-801, myo5∆::natNT2, SAC6-EGFP::his3MX6, ABP1-mCherry::kanMX4 |
| **MKY3871** | MATa, his3-Δ200, leu2-3,112, ura3-52, lys2-801, myo3∆::natNT2, MYO5-EGFP::his3MX6 |
| **MKY3884** | MATa/α, his3-Δ200/his3-Δ200, leu2-3,112/leu2-3,112, ura3-52/ura3-52, lys2-801/lys2-801, MYO5-EGFP::his3MX6/MYO5-EGFP::his3MX6 |
| **MKY3892** | MATa/α, his3-Δ200/his3-Δ200, leu2-3,112/leu2-3,112, ura3-52/ura3-52, lys2-801/lys2-801, MYO5-EGFP::his3MX6/myo5∆::natNT2 |
| **MKY3907** | MATa, his3-Δ200, leu2-3,112, ura3-52, lys2-801, MYO5-EGFP::his3MX6::hphNT1::MYO5-EGFP::his3MX6, myo3∆::natNT2 |
| **MKY3987** | MATa, his3-Δ200, leu2-3,112, ura3-52, lys2-801, myo5∆::natNT2, ABP1-mCherry::kanMX4, pMK0100[CEN, URA3 GFP-ACT1] |
| **MKY4097** | MATa, his3200, leu2-3,112, ura3-52, lys2-801, myo5-G132R::URA, Sla1-EGFP::HISMX6 |
| **MKY4099** | MATa, his3200, leu2-3,112, ura3-52, lys2-801, myo5-G132R-EGFP::URA |
| **JRY0009** | MATα, his3-Δ200, leu2-3,112, ura3-52, lys2-801, ABP1-mMaple::HIS3MX6 |
| **JRY0020** | MATα, his3-Δ200, leu2-3,112, ura3-52, lys2-801, ARC18-mMaple::HIS3MX6 |
| **JRY0024** | MATα, his3-Δ200, leu2-3,112, ura3-52, lys2-801, CAP1-mMaple::HIS3MX6 |
| **JRY0086** | MATα, his3-Δ200, leu2-3,112, ura3-52, lys2-801, ARC18-mMaple::his3MX6, myo5Δ::natNT2 |
| **JRY0087** | MATa, his3-Δ200, leu2-3,112, ura3-52, lys2-801, ABP1-mMaple::his3MX6, myo5Δ::natNT2 |
| **JRY0090** | MATα, his3-Δ200, leu2-3,112, ura3-52, lys2-801, CAP1-mMaple::his3MX6, myo5Δ::natNT2 |

## Supplemental table 4: Plasmids used in this work

| **Name** | **Original name** | **Tag** | **Selection** | **Origin** |
| --- | --- | --- | --- | --- |
| **pMK0003** | pFA6a-EGFP-HIS4MX | EGFP | his3MX6 | Janke, 2004 |
| **pMK0005** | pFA6a-mCherry-KanMX4 | mCherry | kanMX4 | Janke, 2004 |
| **pMK0019** | pFA6a-natNT2 | - | natNT2 | Janke, 2004 |
| **pMK0052** | pks133 | - | hphNT1 | Janke, 2004 |
| **pMK0075** | pYM12-PKS134 | myEGFP | natNT2 | Boeke, 2014 |
| **pMK0088** | pMaM173 | sfGFP | URA | Khmelinskii, 2011 |
| **pMK0100** | pDD302 | GFP-Act1 | URA | Kaksonen, 2003 |
| **pJR58** | pFA6a-mMaple-HIS3MX6 | mMaple | his3MX6 | Mund, 2018 |

|  |  | **Single-color trajectories used to calculate average trajectory** | | | **dual-color trajectory pairs used for alignment** | | |
| --- | --- | --- | --- | --- | --- | --- | --- |
| **Genotype** | **Protein** | **single-color strain** | **nr cells** | **nr single-color trajectories** | **dual-color strain** | **nr cells** | **nr dual-color trajectory pairs** |
| WT | Sla1 | MKY2833 | 13, 10 | 37, 34 | MKY3074 | 14 | 60 |
| WT | Sla2 | MKY2689 | 14 | 30 | MKY2836 | 17 | 48 |
| WT | Rvs167 | MKY2832 | 15 | 112 | MKY3131 | 16 | 43 |
| WT | Abp1 | MKY2834 | 12 | 37 | NA | NA | NA |
| WT | Cap1 | MKY2865 | 16 | 35 | MKY3660 | 16 | 44 |
| WT | Arc18 | MKY2747 | 10 | 31 | MKY2720 | 14 | 46 |
| WT | Sac6 | MKY3863 | 13 | 39 | MKY3865 | 15 | 47 |
| WT | Act1 | MKY2654 | 12 | 47 | MKY2655 | 9 | 31 |
| WT | Myo3 | NA | NA | NA | MKY3459 | 13 | 39 |
| WT | Myo5 | NA | NA | NA | MKY3151 | 14 | 43 |
| myo5Δ | Sla1 | MKY3166 | 12, 12 | 28, 25 | MKY3186 | 16 | 40 |
| myo5Δ | Sla2 | MKY3616 | 16 | 31 | MKY3632 | 16 | 37 |
| myo5Δ | Rvs167 | MKY3228 | 16 | 32 | MKY3229 | 13 | 36 |
| myo5Δ | Abp1 | MKY3188 | 16 | 44 | NA | NA | NA |
| myo5Δ | Cap1 | MKY3565 | 12 | 25 | MKY3658 | 14 | 42 |
| myo5Δ | Arc18 | MKY3665 | 17 | 39 | MKY3667 | 14 | 44 |
| myo5Δ | Sac6 | MKY3867 | 12 | 45 | MKY3868 | 14 | 46 |
| myo5Δ | Act1 | MKY3590 | 9 | 16 | MKY3987 | 10 | 27 |
| myo3Δ | Sla1 | MKY3216 | 14, 16 | 34, 42 | x | x | x |
| MYO5 dupl. | Sla1 | MKY3508 | 13, 13 | 32, 36 | x | x | x |
| WT/WT | Sla1 | MKY3173 | 21, 15 | 49, 34 | x | x | x |
| MYO5/myo5Δ | Sla1 | MKY3186 | 16, 15 | 44, 42 | x | x | x |
| myo5Δ/myo5Δ | Sla1 | MKY3175 | 18, 15 | 49, 37 | x | x | x |
| myo5-CAΔ | Sla1 | MKY3355 | 14, 13 | 29, 33 | x | x | x |
| bbc1Δ | Sla1 | MKY3412 | 9 | 22 | x | x | x |
| myo5Δ bbc1Δ | Sla1 | MKY3815 | 7 | 21 | x | x | x |

## Supplemental table 5: Number of tracked endocytic sites per dataset.

For several strains independent replicates were obtained; the N-values for each replicate are reported, separated by a comma.

| Genotype | Protein | Strain | Nr of analyzed target cells | Nr of analyzed endocytic patches | Nr of analyzed Nuf2 cells | Nr of analyzed Nuf2 spots | Median nr of molecules over the lifetime of the patch | SEM |
| --- | --- | --- | --- | --- | --- | --- | --- | --- |
| WT | Abp1 | MKY2834 | 40 | 327 | 21 | 42 | 282.7 | 29.9 |
| myo5Δ | Abp1 | MKY3188 | 93 | 548 | 45 | 90 | 245.2 | 25.9 |
| WT/WT | Abp1 | MKY3226 | 64 | 495 | 31 | 62 | 260.7 | 27.5 |
| WT | Act1 | MKY2653 | 37 | 187 | 28 | 56 | 2145.6 | 336.7 |
| myo5Δ | Act1 | MKY3590 | 40 | 184 | 23 | 46 | 1305.6 | 210.3 |
| myo5-CAΔ | Act1 | MKY3754 | 36 | 205 | 31 | 62 | 2303.3 | 357.8 |
| bbc1Δ | Act1 | MKY2990 | 45 | 262 | 41 | 82 | 5122.2 | 795.3 |
| myo5Δ bbc1Δ | Act1 | MKY3819 | 28 | 181 | 22 | 44 | 2244.4 | 358.7 |
| WT | Arc18 | MKY2747 | 57 | 274 | 43 | 86 | 127.9 | 29.7 |
| myo5Δ | Arc18 | MKY3665 | 37 | 156 | 37 | 74 | 90.4 | 21.3 |
| myo5-CAΔ | Arc18 | MKY3733 | 49 | 249 | 33 | 66 | 93.2 | 21.8 |
| WT | Cap1 | MKY2865 | 35 | 218 | 24 | 48 | 101.6 | 11.1 |
| myo5Δ | Cap1 | MKY3656 | 41 | 194 | 27 | 54 | 80.6 | 8.7 |
| WT | Myo3 | MKY3318 | 58 | 170 | 37 | 74 | 37.8 | 4.0 |
| myo5Δ | Myo3 | MKY3340 | 45 | 127 | 29 | 58 | 34.1 | 3.8 |
| MYO3 dupl. | Myo3 | MKY3634 | 48 | 251 | 39 | 78 | 59.5 | 6.3 |
| MYO3 dupl. myo5Δ | Myo3 | MKY3565 | 42 | 246 | 38 | 76 | 56.7 | 6.1 |
| MYO5 dupl. | Myo3 | MKY3535 | 51 | 151 | 34 | 68 | 40.1 | 4.4 |
| WT/WT | Myo3 | MKY3512 | 46 | 262 | 33 | 66 | 35.1 | 3.8 |
| MYO5/myo5Δ | Myo3 | MKY3513 | 34 | 245 | 27 | 54 | 36.3 | 3.9 |
| myo5Δ/myo5Δ | Myo3 | MKY3514 | 36 | 243 | 24 | 48 | 34.9 | 3.8 |
| MYO3/myo3Δ myo5Δ/myo5Δ | Myo3 | MKY3526 | 46 | 195 | 37 | 74 | 14.4 | 1.6 |
| WT | Myo5 | MKY2876 | 59 | 302 | 38 | 76 | 76.7 | 8.1 |
| myo3Δ | Myo5 | MKY3871 | 35 | 160 | 22 | 44 | 87.2 | 9.6 |
| MYO3 dupl. | Myo5 | MKY3633 | 50 | 279 | 44 | 88 | 74.9 | 8.0 |
| MYO5 dupl. | Myo5 | MKY3533 | 35 | 165 | 18 | 36 | 111.4 | 11.9 |
| MYO5 dupl. myo3Δ | Myo5 | MKY3907 | 31 | 113 | 18 | 36 | 156.3 | 16.8 |
| WT/WT | Myo5 | MKY3884 | 41 | 99 | 33 | 66 | 76.6 | 8.3 |
| MYO5/myo5Δ | Myo5 | MKY3892 | 26 | 75 | 18 | 36 | 58.0 | 6.7 |
| WT | Rvs167 | MKY2832 | 31 | 99 | 20 | 40 | 55.5 | 6.3 |
| myo5Δ | Rvs167 | MKY3227 | 44 | 64 | 37 | 74 | 47.4 | 5.7 |
| WT | Sac6 | MKY3863 | 29 | 156 | 20 | 40 | 237.5 | 25.1 |
| myo5Δ | Sac6 | MKY3867 | 28 | 151 | 20 | 40 | 160.5 | 18.7 |
| WT | Sla1 | MKY2833 | 85 | 345 | 44 | 88 | 53.0 | 5.9 |
| myo5Δ | Sla1 | MKY3166 | 77 | 401 | 35 | 70 | 53.4 | 5.7 |
| WT/WT | Sla1 | MKY3173 | 45 | 323 | 32 | 64 | 59.6 | 6.4 |
| WT | Sla2 | MKY2689 | 37 | 148 | 29 | 58 | 29.5 | 3.5 |
| myo5Δ | Sla2 | MKY3616 | 42 | 200 | 29 | 58 | 29.5 | 3.4 |

## Supplemental table 6: Number of analyzed endocytic sites for quantification of molecule numbers

| Tomogram | Sla1-EGFP, Abp1-mCherry? | Structure | Invagi-nation length (nm) | Invagi-nation tip diameter (nm) | Vesicle long axis (nm) | Vesicle short axis (nm) | Notes |
| --- | --- | --- | --- | --- | --- | --- | --- |
| 1_H4_3spot5A | Only Sla1-EGFP | Flat PM |  |  |  |  |  |
| 1_N1_1spot4C | Only Sla1-EGFP | Flat PM |  |  |  |  |  |
| 1_N2_2spot1C | Only Abp1-mCherry | invagination | 20 | NA |  |  | dimple |
| 1_H4_3spot5B | Sla1-EGFP and Abp1-mCherry | 2 invaginations | 58 | 25 |  |  | top invagination |
| "" | "" | "" | 63 | 34 |  |  | bottom invagination |
| 1_H4_1spot1A | Sla1-EGFP and Abp1-mCherry | invagination | 60 | 42 |  |  |  |
| 1_N3_3spot3A | Only Abp1-mCherry | invagination | 63 | 29 |  |  |  |
| 3_B8_3spot5B | Sla1-EGFP and Abp1-mCherry | invagination | 65 | 30 |  |  |  |
| 1_N3_2spot3A | Only Abp1-mCherry | invagination | 71 | 37 |  |  |  |
| 1_N1_1spot4B | Only Abp1-mCherry | invagination | 72 | 39 |  |  |  |
| 1_H4_3spot1B | Only Abp1-mCherry | invagination | 81 | 26 |  |  |  |
| 1_N1_2spot2B | Only Abp1-mCherry | 2 invaginations | 94 | 50 |  |  | top invagination |
| "" | "" | "" | 107 | 38 |  |  | bottom invagination, bended |
| 1_N1_2spot2A | Only Abp1-mCherry | invagination | 129 | 40 |  |  |  |
| 1_M4_3spot1A | Only Abp1-mCherry | vesicle |  |  | 42 | 31 |  |
| 1_N2_2spot1A | Only Abp1-mCherry | vesicle |  |  | 53 | 48 |  |
| 1_H4_3spot1A | Only Abp1-mCherry | 2 vesicles |  |  | 45 | 29 | bottom right vesicle |
| "" | "" | "" |  |  | 43 | 34 | top left vesicle |
| 3_B8_3spot5A | Only Abp1-mCherry | vesicle |  |  | 54 | 23 |  |

## Supplemental table 7: Summary of CLEM data
